# Expression of FOXA1 and GATA-3 in breast cancer: the prognostic significance in hormone receptor-negative tumours

**DOI:** 10.1186/bcr2327

**Published:** 2009-06-23

**Authors:** André Albergaria, Joana Paredes, Bárbara Sousa, Fernanda Milanezi, Vítor Carneiro, Joana Bastos, Sandra Costa, Daniella Vieira, Nair Lopes, Eric W Lam, Nuno Lunet, Fernando Schmitt

**Affiliations:** 1 Development Domain, Institute of Life and Health Sciences (ICVS), School of Health Sciences of Minho University – Campus de Gualtar, Braga 4710-057, Portugal; 2 Cancer Genetics Group, Institute of Molecular Pathology and Immunology of Porto University (IPATIMUP), Rua Dr Roberto Frias s/n, Porto 4200-465, Portugal; 3 Department of Pathology of Hospital of Divino Espírito Santo, Rua da Grotinha, Ponta Delgada 9500-370, Portugal; 4 Department of Hygiene and Epidemiology, University of Porto Medical School, Alameda Prof. Hernâni Monteiro, Porto 4200-319, Portugal; 5 Institute of Public Health of the University of Porto (ISPUP), Praça Gomes Teixeira s/n, Porto 4099-002, Portugal; 6 Department of Pathology, Federal University of Santa Catarina, Campus Reitor João David Ferreira Lima, Florianópolis, Santa Catarina CEP 88040-970, Brazil; 7 Department of Oncology, Cancer Research UK Laboratories, MRC Cyclotron Building, Imperial College of London, Hammersmith Hospital, Du Cane Road, London W12 0HS, UK; 8 Department of Pathology, Medical Faculty of University of Porto, Alameda Prof. Hernâni Monteiro, Porto 4200-319, Portugal

## Abstract

**Introduction:**

The expression of additional genes, other than oestrogen receptor (ER), may be important to the hormone-responsive phenotype of breast cancer. Microarray analyses have revealed that forkhead box A1 (FOXA1) and GATA binding protein 3 (GATA-3) are expressed in close association with ERα, both encoding for transcription factors with a potential involvement in the ERα-mediated action in breast cancer. The purpose of this study was to explore if the expression of FOXA1 and GATA-3 may provide an opportunity to stratify subsets of patients that could have better outcome, among the ERα-negative/poor prognosis breast cancer group.

**Methods:**

We evaluate FOXA1 and GATA-3 expression in 249 breast carcinomas by immunohistochemistry, associating it with breast cancer molecular markers, clinicopathological features and patient's survival. The clinicopathological features and immunohistochemical markers of the tumours were compared using the chi-square test and ANOVA. Disease-free survival was analysed through Kaplan–Meier survival curves and Cox regression.

**Results:**

FOXA1 expression was demonstrated in 42% of invasive carcinomas, while GATA-3 was detected in 48% of the cases. FOXA1 expression was inversely associated with tumour size, Nottingham Prognostic Index, histological grade, lymph vascular invasion, lymph node stage and human epidermal growth factor receptor-2 (HER-2) overexpression, while GATA-3 expression showed inverse association with histological grade and HER-2. Both FOXA1 and GATA-3 were directly associated with ERα and progesterone receptor. Among FOXA1-positive tumours, 83.1% are comprised in the luminal A subtype, similar to GATA-3 where 87.7% of positive tumours were classified within this molecular subtype. In the subset of ERα-negative patients, those who were FOXA1-negative had a 3.61-fold increased risk of breast cancer recurrence when compared with the FOXA1-positive.

**Conclusions:**

FOXA1 was a significant predictor of good outcome in breast cancer, whereas GATA-3 was an important luminal marker. The expression of FOXA1 may be used for risk stratification among ERα-negative patients.

## Introduction

The expression of oestrogen receptor (ER) is an important prognostic and predictive factor in breast cancer and has relevant implications for the biology of this type of carcinomas. Patients with tumours that express ER have a longer disease-free interval and overall survival than patients with tumours lacking ER expression [1].

According to international treatment guidelines for early breast cancer, patients with ERα and/or progesterone receptor (PR) expression should receive an adjuvant endocrine therapy, since their expression is associated with higher response rates to anti-hormonal treatment [2]. However, the association between ERα expression and hormonal responsiveness is far from perfect, since approximately 30% of ER-positive tumours do not respond to hormonal treatment and 5 to 15% of ER-negative tumours curiously respond to endocrine therapy [3].

In order to overcome and explore this unpredictable breast tumour behaviour, numerous studies, based on cDNA microarrays, have shown that the gene expression profile in breast cancer can provide molecular phenotypes that identify distinct tumour subclasses [4-6], patient survival prediction [5-7], and differences in tumour biology or clinical features. The molecular classification of breast cancers distinguishes three major subtypes: the ER-positive/luminal-like subtype, a gene expression cluster characteristic of the luminal cells and anchored by a cluster of transcription factors that include ER; the basal-like subtype, comprising tumours that express basal cell markers (namely keratin 5, keratin 14, integrin β_4 _and laminin); and the human epidermal growth factor receptor-2 (HER-2)-overexpressing subtype, usually associated with gene amplification of the HER-2 proto-oncogene and high expression of several genes in the ERBB2 amplicon at 17q22.24 [4,5,8]. These studies have largely contributed to understanding the complex behaviour of certain types of breast cancer, including the ones that respond better to endocrine therapies, regardless of ER expression.

Oestrogen plays an important role in the regulation of growth, proliferation and differentiation of mammary epithelium. The action of oestrogen is mediated through the ER, which functions as an oestrogen-activated transcription factor. The expression of an additional set of genes that is not part of the canonical oestradiol-response pathway may also be essential in clarifying the hormone-responsive phenotype, since intrinsic differences in the list of transcription factors bound to the *ER *gene promoter have been described [9].

Additionally, the distinct behaviour observed between ER-positive luminal subtypes A and B (a subgroup of tumours with low to moderate expression of the luminal-specific genes including the ER cluster) may in part be due to the influence of additional factors, including transcriptional factors, co-activators and co-repressors modulating ER activity [10], which can also be explored towards a therapeutic purpose.

In 2004 Lacroix and Leclercq compiled considerable extensive data describing the strong association and cross-talk between ERα, forkhead box A1 (FOXA1) and GATA binding protein 3 (GATA-3) [11]. In most of these studies, GATA-3 and FOXA1 have been highlighted within the ERα pathway in the luminal A subtype [4-6,12]; FOXA1, a forkhead family transcription factor, has been receiving considerable attention, since it interacts with *cis*-regulatory regions of heterochromatin, enhancing the interaction of ERα with DNA [13]. Carroll and colleagues recently described several robust data demonstrating the requirement of FOXA1 for optimal expression of nearly 50% of ERα-regulated genes and oestrogen-induced proliferation [13,14].

FOXA1 is expressed in the liver, pancreas, bladder, prostate, colon and lung, as well as in the mammary gland, and can bind to the promoters of more than 100 genes associated with metabolic processes, regulation of signalling pathways and cell cycle [15-17]. Some studies have shown that FOXA1 can act either as a growth stimulator/activator or as a repressor. As a stimulator, FOXA1 binds to chromatinised DNA and opens the chromatin, enhancing binding of ERα to its target genes [18] – which suggests a growth-promoting role for this forkhead protein [14,18]. In breast cancer, however, FOXA1 overexpression can also block the metastatic progression by influencing the expression of the BRCA1-associated cell-cycle inhibitor p27 and promoting E-cadherin expression [19,20]. Recent studies also suggest FOXA1 as a favourable prognostic factor in breast cancer, with potential relevance in the subclassification of luminal/ER-positive tumours into two subgroups with different biological behaviour and prognosis, the luminal A and the luminal B [5].

FOXA1 and ERα have been explored as potential participants involved in mammary tumours together with another gene, GATA-3 [21,22], which regulates the lineage determination and differentiation of many cells types. In the breast, GATA-3 plays a central role in luminal epithelia differentiation and the subsequent formation of the ductal tree of differentiated epithelial cells [23], suggesting that this protein might be involved in breast tumorigenesis [24].

Meta-analysis of four microarray datasets indicated that GATA-3 was a strong predictor of clinical outcome in breast tumours and is among the best predictors of ER-positive status [4,9,25-27]. Among all of the molecular subgroups of breast cancer, the luminal A subtype has a relatively favourable outcome and the highest GATA-3 and ERα expression levels, compared with luminal B and basal-like breast carcinomas [24].

As a result of all of these extensive studies underlying GATA-3 and ERα in mammary epithelia, it has been clear that GATA-3 is a crucial regulator of tumour differentiation and suppressor of tumour dissemination [22]. It has been also suggested that these functions in mammary luminal cells may be linked by transcriptional regulators, whereas FOXA1 appears as a candidate gene, which is necessary for the transcriptional activity of ERα and its binding to oestrogen-responsive elements in target gene promoters [13,18]. As FOXA1 may also be a downstream effector of GATA-3, it may be a bridge between GATA-3 and ER pathways [22], controlling and regulating the biology of luminal mammary cells, breast cancer progression and behaviour.

Based on this intricate and functional complex between FOXA1 and GATA-3 in breast cancer biology, it is reasonable to consider that these transcription factors, in addition to ERα, are important in establishing and clarifying the hormone-responsive phenotype and prognosis in breast cancer. This gene set may therefore be used as a diagnostic tool for more accurate determination of ERα status, in the decision on endocrine therapeutic strategies, as well as in the assessment of breast cancer patient's outcome.

In the present study we provide an immunohistochemical approach studying FOXA1 and GATA-3 expression, in order to predict the tumour behaviour of breast cancer patients. In the whole series, we verified that patients harbouring FOXA1-positive tumours show a better disease-free survival. Interestingly, and for the first time, we also found the same power of risk stratification among the ERα-negative breast cancer patients, demonstrating the clinical importance of this biomarker in breast cancer molecular classification and prognosis. These results show that FOXA1 and ERα should be used together in order to subclassify breast carcinomas and to predict the outcome of breast cancer patients.

## Materials and methods

### Patient selection

A series of 249 cases of primary operable invasive breast carcinomas were retrieved from the files of the Department of Pathology, Hospital do Divino Espírito Santo, Azores, Portugal and from the Federal University of Santa Catarina, Florianopolis – SC, Brazil. These samples were obtained from patients with age ranging from 30 to 89 years. All of the formalin-fixed paraffin-embedded histological sections were reviewed by three pathologists (VC, FS and FM) and the diagnoses were confirmed as follows: 208 invasive ductal carcinomas, seven invasive lobular carcinomas, three mixed breast carcinomas, three tubular breast carcinomas, eight medullary breast carcinomas and 20 invasive breast carcinomas of other special histological types. These tumours have been fully characterized for clinical and pathological features – namely, age, tumour size, histological type, lymph nodes invasion, tumour grade, Nottingham Prognostic Index, ERα, PR and HER-2 status. The patients' clinical and pathological characteristics are summarized in Table 1.

**Table 1:** Patient characteristics and tumour parameters

Variable	Data
Age at diagnosis (years)	
Mean and standard deviation	57 ± 14.2
Range	59 (minimum 30; maximum 89)
Tumour size (mm)	
Mean and standard deviation	32 ± 21 mm
Range	145 (minimum 5; maximum 150)
Lymphovascular invasion	
Present	111 (44.6)
Absent	111 (44.6)
Not assessed	27 (10.8)
Lymph node sage	
Negative	111 (44.6)
1 to 3 lymph nodes	57 (22.9)
>3 lymph nodes	54 (21.7)
Not assessed	27 (10.8)
Tumour grade	
Grade I	51 (20.5)
Grade II	116 (46.6)
Grade III	82 (32.9)
Histology	
Invasive ductal carcinoma (not otherwise specified)	208 (83.5)
Invasive lobular carcinoma	7 (2.8)
Mixed	3 (1.2)
Tubular	3 (1.2)
Medullary	8 (3.3)
Other special types	20 (8.0)
Nottingham Prognostic Index	
<3.4	46 (18.5)
3.4 to 5.4	106 (42.6)
>5.4	55 (22.0)
Not assessed	42 (16.9)
Oestrogen receptor-α status	
Positive	141 (56.6)
Negative	107 (43.0)
Unknown	1 (0.4)
Progesterone receptor status	
Positive	89 (35.8)
Negative	154 (61.8)
Unknown	6 (2.4)
Human epidermal growth factor receptor 2 status	
Positive	42 (16.9)
Negative	201 (80.7)
Unknown	6 (2.4)

Data presented as *n *(%) unless stated otherwise.

Follow-up information was available for 218 cases, ranging from a minimum of 2 months to a maximum of 129 months (median 32 months). The disease-free survival data interval was evaluated and defined as the time from the date of surgery to the date of breast-cancer-derived relapse/metastasis. Owing to the short follow-up of the studied series and the consequent limited number of death events, overall survival was not analysed.

The present study was conducted under the national regulative law for the usage of biological specimens from tumour banks, where the samples are exclusively available for research purposes in the case of retrospective studies.

### Tissue microarray construction and immunohistochemical analysis

Representative areas of different lesions were carefully selected on haematoxylin and eosin-stained sections and were marked on individual paraffin blocks. Two tissue cores (2 mm in diameter) were obtained from each selected specimen and were precisely deposited into a recipient paraffin block using a tissue microarray workstation (tissue microarray builder ab1802; Abcam, Cambridge, UK) as described elsewhere [28,29]. In each tissue microarray block, non-neoplastic breast tissue cores were also included as controls.

Immunohistochemistry was performed in 3 μm formalin-fixed, paraffin-embedded sections. Expression for the most commonly used breast cancer markers – namely, HER-2, ER, PR, P-cadherin, epidermal growth factor receptor (EGFR), vimentin and basal cytokeratins (CK5, CK14) – was assessed. The immunohistochemistry technique was performed using an Envision Detection System (DAKO Cytomation Envision System HRP; DAKO Corporation, Carpinteria, CA, USA) or the classical streptavidin – avidin – biotin complex method according to the manufacturer's instructions. Imunohistochemistry assay conditions and antibodies specifications were based on previously published studies from our group [28-30]. Immunoreactivity for ERα, PR, P-cadherin, CK5, CK14, EGFR, vimentin and HER-2 was classified by estimating the percentage of tumour cells showing characteristic staining, in accordance with previous work [28-30].

Expression of FOXA1 was analysed using a mouse monoclonal antibody (clone 2F83, ab40868; AbCam), as well as GATA-3 expression (clone H-63-31, Sc-268; Santa Cruz Biotechnology Inc., Santa Cruz, CA, USA). Sections were deparaffinized with xylene and rehydrated in a series of decreasing concentration of ethanol solutions. Heat-induced epitope retrieval was carried out in 10 mM citrate buffer (sodium citrate) (pH 6) or in 1 mM ethylenediamine tetraacetic acid buffer (pH 8) (LabVision Corporation, Fremont, CA, USA), in a 98°C water bath, for 14 and 20 minutes for FOXA1 and GATA-3, respectively. After cooling retrieval solutions for at least 30 minutes at room temperature, the slides were treated for 10 minutes with 3% H_2_O_2 _in methanol, in order to block endogenous peroxidase. Slides were incubated with monoclonal antibodies for FOXA1 (1:450) and GATA-3 (1:100) for 2 hours at room temperature and were labelled with the Envision Detection System from DAKO. Colour reaction product was developed with 3,3'-diaminobenzidine, tetrahydrochloride (DAB plus; DAKO Glostrup, Denmark) as a substrate, and nuclear contrast was achieved with haematoxylin/ammoniacal water counterstaining. Formalin-fixed, paraffin-embedded sections from normal breast gland were used as FOXA1 and GATA-3 positive controls. Negative controls were performed by replacing the primary antibody with PBS/nonimmune mouse serum.

The scoring method used for FOXA1 and GATA-3 expression was based on a semi-quantitative scoring system previously described by Thorat and colleagues, where the cutoff value for FOXA1 positivity was validated [31]. In this scoring system, the percentage of staining was categorized as: 0 = no nuclear expression; 1 = 1 to 10% positive tumour nuclei; 2 = 11 to 20%; and so on until a maximum score of 10 = 91 to 100% positive tumour nuclei. The intensity was scored as: 1+ = weak staining; 2+ = moderate staining; and 3+ = strong staining. The numeric final score was generated by the multiplication product of percentage and intensity of nuclear expression (scoring = percentage × intensity) [10,32]. Based on this semiquantitative scoring system, scores between 0 and 3 were classified as negative, and scores ≥ 4 to a maximum of 30 were considered positive.

### Statistical analysis

Statistical analysis was performed using Stata™, version 9.2 software (StataCorp, College Station, TX, USA). Continuous variables were presented as the mean ± standard deviation, and categorical variables were presented as the number (percentage). The clinicopathological features and immunohistochemical markers of the tumours were compared across groups of expression of FOXA1 and GATA-3 using analysis of variance and the chi-square test, respectively, for continuous and categorical variables.

Survival curves were estimated by the Kaplan – Meier method using the log-rank test to assess significant differences for disease-free patient survival. A maximum cutoff value of 60 months (5 years) was considered, since this is the expected clinical time for breast cancer recurrence. Cox regression models were fitted to estimate hazard ratios and the corresponding 95% confidence interval for the classical prognostic factors, FOXA1 and GATA-3. In all analyses, a significant level of 5% was considered.

## Results

### FOXA1 and GATA-3 expression in normal and malignant breast tissues

**Figure 1:**
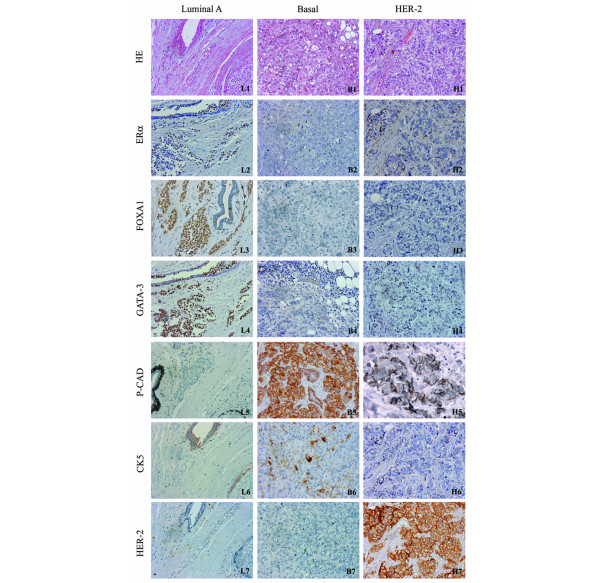
Immunohistochemistry panel showing differential expression pattern of FOXA1 and GATA-3. An example of luminal A **(L1 to L7)**, basal-like **(B1 to B7) **and human epidermal growth factor receptor 2 (HER-2)-overexpressing **(H1 to H7) **invasive breast tumours. Expression of the most commonly used breast cancer markers is also illustrated for comparison with the forkhead box A1 (FOXA1) and GATA binding protein 3 (GATA-3) expression. (L1, B1, H1) Haematoxylin-eosin stainings from each of the selected core cases. (L3, L4) Strong and restricted nuclear expression of FOXA1 and GATA-3 in the normal breast duct (internal control) and in the luminal A invasive tumour (grade II). (B3, B4) Negative expression of FOXA1 and GATA-3 in basal subtype tumour (grade III). (H3, H4) HER-2-overexpressing tumour showing negativity for FOXA1 and GATA-3 expression (grade III). All microscopy images are at 40× magnification. ER, oestrogen receptor; P-CAD, P-cadherin; CK, cytokeratin.

From the total 249 cases, only cases with clear and restricted nuclear expression for FOXA1 and GATA-3 were selected for immunohistochemistry classification. Three representative cases were selected to build a panel, illustrated in Figure 1, comprising a classical example of the following molecular subtypes of breast cancer: luminal A subtype (Figure 1, L1 to L7), basal-like subtype (Figure 1, B1 to B7) and HER-2-overexpressing subtype (Figure 1, H1 to H7). Strong immunoexpression of FOXA1 and GATA-3 in the nuclei of malignant cells, as well as in some luminal epithelial cells from adjacent normal ducts, is shown in Figure 1 (L3 and L4). FOXA1 was positive (score ≥ 4) in the nuclei of 42% (93 out of 224) of the invasive carcinomas, while GATA-3 was detected in 48% (97 out of 204) of the cases.

### Association between FOXA1 and GATA-3 expression and clinicopathological features and biological markers

The expression of FOXA1 was inversely associated with tumour size (*P *= 0.005), Nottingham Prognostic Index (*P *= 0.002), histological grade (*P *= 0.001), vascular invasion (*P *= 0.012), lymph node stage (*P *= 0.022) and HER-2 overexpression (*P *= 0.017), and was directly associated with ERα expression (*P *< 0.0001) and PR expression (*P *< 0.0001). GATA-3 expression showed an inverse association with histological grade (*P *= 0.013) and HER-2 overexpression (*P *< 0.0001), and a direct association with ERα expression (*P *< 0.001) and PR expression (*P *< 0.001) (Table 2).

**Table 2:** Association between FOXA1 and GATA3 expression and the clinicopathological features of the infiltrative breast carcinomas

Variable	*N*	FOXA1-negative (%)	FOXA1-positive (%)	*P *value	*n*	GATA3-negative (%)	GATA3-positive (%)	*P *value
Tumour size (mm)	209	35.2 ± 24.0 (126)	26.8 ± 15.7 (83)	0.005	191	34.4 ± 25.2 (100)	28.6 ± 16.7 (91)	0.064
Lymphovascular invasion	203				185			
Present	97	67 (69.1)	30 (30.9)	0.012	92	49 (53.3)	43 (46.7)	0.829
Absent	106	55 (51.9)	51 (48.1)		93	51 (54.8)	42 (45.2)	
Lymph node stage	203				185			
Negative	106	55 (51.9)	51 (48.1)	0.022	93	51 (54.8)	42 (45.2)	0.166
1 to 3 lymph nodes	49	31 (63.3)	18 (36.7)		46	20 (43.5)	26 (56.5)	
>3 lymph nodes	48	36 (75)	12 (25)		46	29 (63)	17 (37)	
Grade	224				204			
Grade I	44	25 (56.8)	19 (43.2)	0.001	40	24 (60)	16 (40)	0.013
Grade II	105	50 (47.6)	55 (52.4)		96	40 (41.7)	56 (58.3)	
Grade III	75	56 (74.7)	19 (25.3)		68	43 (63.2)	25 (36.8)	
Histology	224				204			
IDC	188	113 (60.1)	75 (39.9)	0.119	171	91 (53.2)	80 (46.8)	0.104
ILC	6	2 (33.3)	4 (66.7)		6	0 (0)	6 (100)	
Tubular	1	1 (100)	0 (0)		1	0 (0)	1 (100)	
Medullary	7	5 (71.4)	2 (28.6)		7	5 (71.4)	2 (28.6)	
Other	19	7 (36.8)	12 (63.2)		16	9 (56.2)	7 (43.8)	
Mixed	3	3 (100)	0 (0)		3	2 (66.7)	1 (33.3)	
Nottingham Prognostic Index	190				172			
<3.4	44	18 (40.9)	26 (59.1)	0.002	40	19 (47.5)	21 (52.5)	0.293
3.4 to 5.4	96	59 (61.5)	37 (38.5)		86	44 (51.2)	42 (48.8)	
>5.4	50	38 (76)	12 (24)		46	29 (63)	17 (37)	
ERα	224				204			
Positive	133	60 (45.1)	73 (54.9)	<0.0001	122	41 (33.6)	81 (66.4)	<0.0001
Negative	91	71 (78)	20 (22)		82	66 (80.9)	16 (19.5)	
PR	223				204			
Positive	83	33 (39.7)	50 (60.3)	<0.0001	74	22 (29.7)	52 (70.3)	<0.0001
Negative	140	98 (70)	42 (30)		130	85 (65.4)	45 (34.6)	
HER-2	220				201			
Positive	35	27 (77.1)	8 (22.9)	0.017	34	29 (85.3)	5 (14.7)	<0.0001
Negative	185	103 (55.7)	82 (44.3)		167	77 (46.1)	90 (53.9)	

FOXA-1, forkhead box A1; GATA-3, GATA binding protein 3; IDC, invasive ductal carcinoma (not otherwise specified); ILC, invasive lobular carcinoma; ERα, oestrogen receptor; PR, progesterone receptor; HER-2, human epidermal growth factor receptor 2.

When we compared the expression of FOXA1 and GATA-3 with the molecular subtype, we found that 83.1% and 87.7% of FOXA1 and GATA-3, respectively, were comprised in the luminal A subtype (*P *< 0.0001) (Table 3 and Figure 1, L1 to L7). Basal-like subtype tumours were negative for FOXA1 (Figure 1, B3) and for GATA-3 (Figure 1, B4) in 85.7% and 84.6% of the cases, respectively (Table 3).

**Table 3:** Association between FOXA1 and GATA3 expression and the immunohistochemical markers in infiltrative breast carcinomas

Variable	*N*	FOXA1-negative (%)	FOXA1-positive (%)	*P *value	*n*	GATA3-negative (%)	GATA3-positive (%)	*P *value
EGFR	223				203			
Positive	13	10 (7.6)	3 (3.3)	0.171	11	11 (10.4)	0 (0)	0.001
Negative	210	121 (92.4)	89 (96.7)		192	95 (89.6)	97 (100)	
P-cadherin	220				202			
Positive	75	53 (40.8)	22 (24.4)	0.012	71	52 (49.1)	19 (19.8)	<0.0001
Negative	145	77 (59.2)	68 (75.6)		131	54 (50.9)	77 (80.2)	
Cytokeratin 5	224				204			
Positive	50	36 (27.5)	14 (15)	0.027	48	39 (36.4)	9 (9.3)	<0.0001
Negative	174	95 (72.5)	79 (85)		156	68 (63.6)	88 (90.7)	
Cytokeratin 14	219				201			
Positive	14	13 (10.1)	1 (1.5)	0.007	14	14 (13.3)	0 (0)	0.0002
Negative	205	116 (89.9)	89 (98.9)		187	91 (86.7)	96 (100)	
Vimentin	203				194			
Positive	34	28 (23.1)	6 (7.3)	0.003	32	26 (25)	6 (6.7)	0.0006
Negative	169	93 (76.9)	76 (92.7)		162	78 (75)	84 (93.3)	
FOXA1	-	-	-	-	201			
Positive	-	-	-		82	16 (15.4)	66 (68)	<0.0001
Negative	-	-	-		119	88 (84.6)	31 (32)	
GATA-3	201				-	-	-	-
Positive	97	31 (26.1)	66 (80.5)	<0.0001	-	-	-	
Negative	104	88 (73.9)	16 (19.5)		-	-	-	
Subtype	202				187			
Luminal A	125	56 (47)	69 (83.1)	<0.0001	114	36 (36.7)	78 (87.7)	<0.0001
Luminal B	8	4 (3.4)	4 (4.8)		8	5 (5.1)	3 (3.4)	
HER-2	27	23 (19.3)	4 (4.8)		26	24 (24.5)	2 (2.2)	
Basal	42	36 (30.3)	6 (7.3)		39	33 (33.7)	6 (6.7)	

FOXA-1, forkhead box A1; GATA-3, GATA binding protein 3; EGFR, epidermal growth factor receptor; HER-2, human epidermal growth factor receptor 2.

The immunohistochemical evaluation of FOXA1 and GATA-3 in breast tumour samples revealed that in 201 of interpretable cases a very significant direct association between the expression of FOXA1 and GATA-3 was observed (*P *< 0.0001) (Table 3).

On the evaluation of these two transcription factors with other important immunohistochemical markers in breast cancer, we found a strong inverse association with basal-like phenotype markers – namely, CK14 (*P *= 0.007, *P *= 0.0002), CK5 (*P *= 0.027, *P *< 0.0001), vimentin (*P *= 0.003, *P *= 0.0006) and P-cadherin (*P *= 0.012, *P *< 0.0001) for FOXA1 and GATA-3, respectively. GATA-3, but not FOXA1, showed an interesting inverse association with EGFR (*P *= 0.001) (Table 3).

### Survival and patient outcome analysis

**Figure 2:**
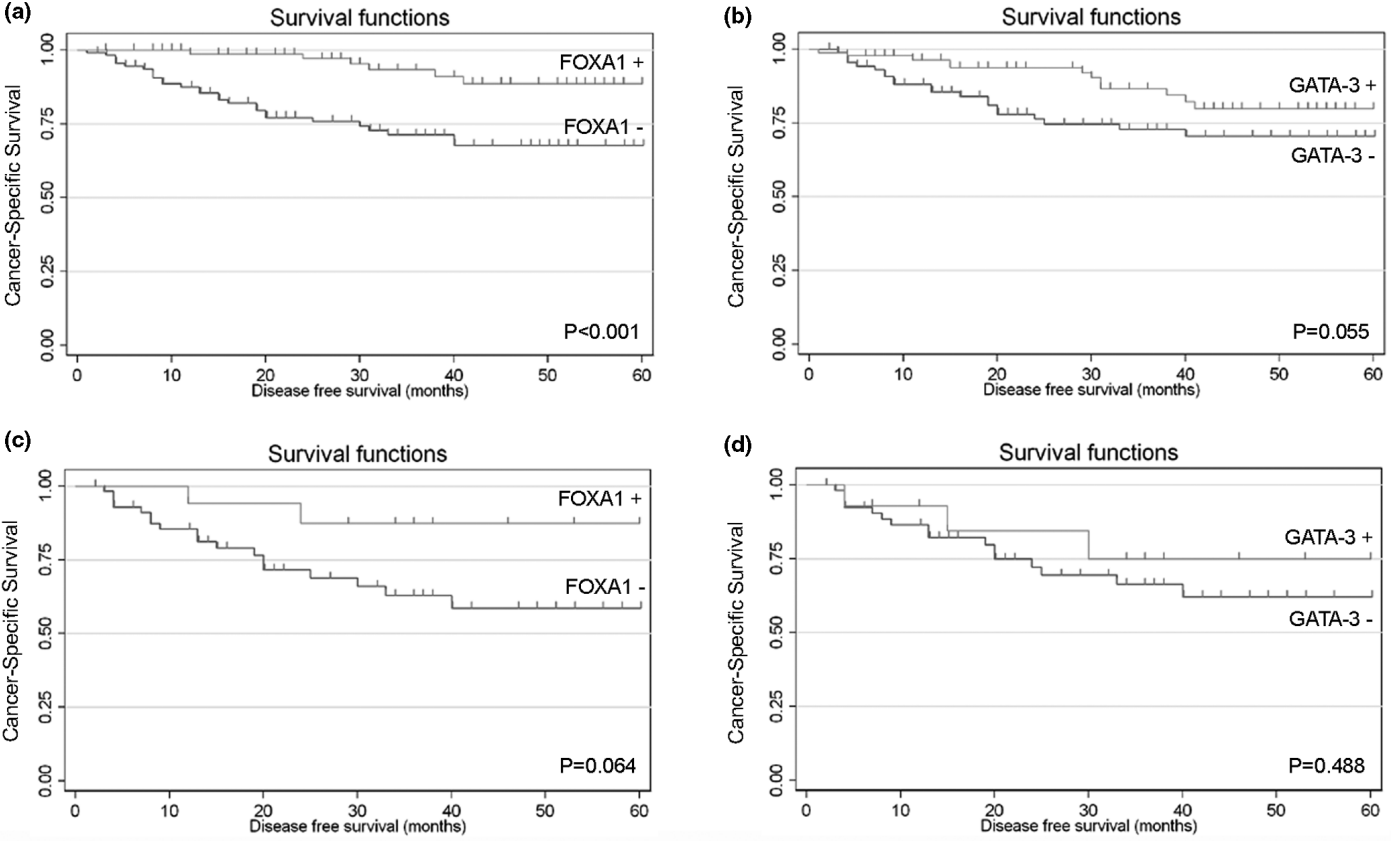
Kaplan–Meier survival curves for disease-free survival. **(a) **Survival functions for forkhead box A1 (FOXA1) in the whole breast cancer patient series (*P *< 0.001). **(b) **Survival functions for GATA binding protein 3 (GATA-3) in the whole breast cancer patient series (*P *= 0.055). **(c) **Survival functions for FOXA1 in the oestrogen receptor α-negative breast cancer patient cohort (*P *= 0.064). **(d) **Survival functions for GATA-3 in the oestrogen receptor α-negative breast cancer patient cohort (*P *= 0.488).

Kaplan–Meier survival curves demonstrate that patients with FOXA1-positive breast carcinomas showed a significant difference towards the longer disease-free survival time (*P *< 0.001; Figure 2a). Although these are no statistically significant differences in survival according to GATA-3 expression (*P *= 0.055; Figure 2b), the positivity for this marker is also associated with a better outcome for breast cancer patients.

As previously demonstrated in other studies, univariate Cox proportional hazard analysis showed that the tumour size, lymph node stage, tumour grade, as well as the expression of ER, PR and HER-2 were significant predictors for disease-free survival. In accordance with the trend shown by the Kaplan–Meier curves, the expression of FOXA1 was also a significant predictor for disease-free survival, showing that negative cases carry a fourfold increased risk of recurrence (hazard ratio = 4.25, 95% confidence interval = 1.76 to 10.28) when compared with the positive ones. In contrast, GATA-3 expression was revealed not to be important as a predictive marker for better outcome in this series (hazard ratio = 1.97, 95% confidence interval = 0.96 to 4.01) (Table 4).

**Table 4:** Univariate Cox proportional hazard analysis (disease-free survival) in the whole breast cancer series

Variable	Evaluation	Hazard ratio (95% confidence interval)	*P *value
Tumour size	T1 (≤ 2 mm)	1	
	T2 (2 < T ≤ 5 mm)	1.57 (0.70 to 3.51)	0.265
	T3 (>5 mm)	3.12 (1.16 to 8.41)	0.024
Lymph node stage	Negative	1	
	1 to 3 lymph nodes	0.56 (0.20 to 1.56)	0.272
	>3 lymph nodes	2.68 (1.30 to 5.49)	0.007
Tumour grade	Grade I	1	
	Grade II	2.60 (0.58 to 11.56)	0.208
	Grade III	7.65 (1.80 to 32.48)	0.006
ER expression	ER-positive	1	
	ER-negative	2.94 (1.52 to 5.57)	0.001
PR expression	PR-positive	1	
	PR-negative	2.16 (1.04 to 4.46)	0.038
HER-2/neu expression	HER-2/neu-negative	1	
	HER-2/neu-positive	2.47 (1.19 to 5.09)	0.014
FOXA1 expression	FOXA1-positive	1	
	FOXA1-negative	4.25 (1.76 to 10.28)	0.001
GATA-3 expression	GATA-3-positive	1	
	GATA-3-negative	1.97 (0.96 to 4.01)	0.061

ER, oestrogen receptor; FOXA-1, forkhead box A1; GATA-3, GATA binding protein 3; HER-2, human epidermal growth factor receptor 2; PR, progesterone receptor.

### Prognostic significance of FOXA1 and GATA-3 expression in ER-negative breast cancer

Several studies have shown that FOXA1 and GATA-3 expression are strong predictors of better clinical outcome in breast tumours and are among the best predictors of ERα-positive status [9-12,26,31,33,34]. Since FOXA1 and GATA-3 show an intrinsic high correlation between themselves and with ERα status, however, the prognostic and predictive value of these markers may simply reflect this high expression association. A cohort of ERα-negative patients was therefore studied in order to evaluate the predictive importance of FOXA1 and GATA-3 expression in this subset of breast carcinomas.

When the association analysis was performed in the subset of ERα-negative patients, FOXA1 and GATA-3 failed to show any significant association with the studied clinicopathological features. Analysing the association of these transcription factors with the immunohistochemical biomarkers in breast cancer, no significant associations were found concerning FOXA1 expression. GATA-3 negativity, however, showed significant association with P-cadherin and CK14 expression. Although not statistically significant, we also observed a trend towards the association of GATA-3 with the other studied basal-like phenotype markers (namely, EGFR, CK5 and vimentin), where the majority of the negative cases for GATA-3 are positive for those proteins (Table 5).

**Table 5:** Association between FOXA1 and GATA3 expression, clinicopathological features and immunohistochemical markers in ER-negative cohort

Variable	*n*	FOXA1-negative (%)	FOXA1-positive (%)	*P *value	*n*	GATA3-negative (%)	GATA3-positive (%)	*P *value
Tumour size	80	36.7 ± 24.2 (65)	36.6 ± 24.8 (15)	0.987	74	34.46 ± 23.6 (60)	43.4 ± 27.4 (14)	0.229
Lymphovascular invasion	83				78			
Present	35	29 (82.8)	6 (17.2)	0.391	35	29 (82.8)	6 (17.2)	0.867
Absent	48	36 (75)	12 (25)		43	35 (81.4)	8 (18.6)	
Lymph node stage	83				78			
Negative	48	36 (75)	12 (25)	0.456	43	35 (81.4)	8 (18.6)	0.609
1 to 3 lymph nodes	12	11 (91.7)	1 (8.3)		12	11 (91.7)	1 (8.3)	
>3 lymph nodes	23	18 (78.3)	5 (21.7)		23	18 (78.3)	5 (21.7)	
Grade	87				80			
Grade I	8	6 (75)	2 (25)	0.166	8	8 (100)	0 (0)	0.366
Grade II	33	23 (69.7)	10 (30.3)		28	22 (78.6)	6 (21.4)	
Grade III	46	40 (86.9)	6 (13.1)		44	36 (81.8)	8 (18.2)	
Nottingham Prognostic Index	76				71			
<3.4	11	9 (81.8)	2 (18.2)	0.982	10	9 (90)	1 (10)	0.615
3.4 to 5.4	34	27 (79.4)	7 (20.6)		32	26 (81.3)	6 (18.7)	
>5.4	31	25 (80.6)	6 (19.4)		29	22 (75.8)	7 (24.2)	
HER-2	85				79			
Positive	26	23 (88.5)	3 (11.5)	0.254	26	24 (92.3)	2 (7.7)	0.102
Negative	59	46 (77.9)	13 (22.1)		53	41 (77.4)	12 (22.6)	
EGFR	86				80			
Positive	12	10 (83.3)	2 (16.7)	0.771	11	11 (100)	0 (0)	0.100
Negative	74	59 (79.7)	15 (20.3)		69	55 (79.7)	14 (20.3)	
P-cadherin	87				80			
Positive	53	45 (84.9)	8 (15.1)	0.107	52	47 (90.4)	5 (9.6)	0.011
Negative	34	24 (70.6)	10 (29.4)		28	19 (67.8)	9 (32.2)	
Cytokeratin 5	87				80			
Positive	33	28 (84.8)	5 (15.2)	0.318	32	29 (90.6)	3 (9.4)	0.118
Negative	54	41 (75.9)	13 (24.1)		48	37 (77.1)	11 (22.9)	
Cytokeratin 14	85				78			
Positive	14	13 (92.8)	1 (7.2)	0.188	14	14 (100)	0 (0)	0.053
Negative	71	55 (77.5)	16 (22.5)		64	50 (78.1)	14 (21.9)	
Vimentin	82				75			
Positive	27	25 (92.6)	2 (7.4)	0.074	25	24 (96)	1 (4)	0.064
Negative	55	42 (76.4)	13 (23.6)		50	40 (80)	10 (20)	
FOXA1	-				78			
Positive	-	-	-	-	15	6 (40)	9 (60)	<0.0001
Negative	-	-	-		63	58 (92.1)	5 (7.9)	
GATA-3	78				-			
Positive	14	5 (35.7)	9 (64.3)	<0.0001	-	-	-	-
Negative	64	58 (90.6)	6 (9.4)		-	-	-	

ER, oestrogen receptor; EGFR, epidermal growth factor receptor; FOXA-1, forkhead box A1; GATA-3, GATA binding protein 3; HER-2, human epidermal growth factor receptor 2.

In this subset of ERα-negative patients, however, an association between loss of FOXA1 expression and worst disease-free survival was found (*P *= 0.064), in contrast with GATA-3 expression (*P *= 0.488) (Figure 2c and 2d, respectively). Moreover, in order to quantify the risk of these survival associations, univariate analysis was performed for FOXA1 and GATA-3 as well as for the classical prognostic factors in breast cancer. In line with the Kaplan–Meier curves, GATA-3 negativity does not account for an increased risk of recurrence in ERα-negative tumours. FOXA1 expression, however, is able to stratify this relative risk among this subset of carcinomas, since its loss accounts for a 3.61-fold increased risk for breast cancer recurrence (Table 6). These results suggest a protective role for this forkhead protein in this poor-outcome breast cancer subgroup.

**Table 6:** Univariate Cox proportional hazard analysis (disease-free survival) in the oestrogen-receptor-negative cohort

Variable	Evaluation	Hazard ratio (95% confidence interval)	*P *value
Tumour size	T1 (≤ 2 mm)	1	
	T2 (2 < T ≤ 5 mm)	1.45 (0.40 to 5.21)	0.567
	T3 (>5 mm)	3.57 (0.88 to 14.4)	0.073
Lymph node stage	Negative	1	
	1 to 3 lymph nodes	0.47 (0.10 to 2.16)	0.338
	>3 lymph nodes	2.49 (1.02 to 6.03)	0.044
Tumour grade	Grade I	^a^	
	Grade II	1	
	Grade III	2.85 (1.05 to 7.57)	0.040
HER-2/neu expression	HER-2/neu-negative	1	
	HER-2/neu-positive	2.04 (0.90 to 4.61)	0.086
FOXA1 expression	FOXA1-positive	1	
	FOXA1-negative	3.61 (0.83 to 15.60)	0.086
GATA-3 expression	GATA-3-positive	1	
	GATA-3-negative	1.53 (0.44 to 5.28)	0.495

FOXA-1, forkhead box A1; GATA-3, GATA binding protein 3; HER-2, human epidermal growth factor receptor 2. ^a^There were no oestrogen-receptor-negative cases classified as grade I among the patients with available follow-up information.

Additionally, the multivariate Cox hazard analysis, with models including tumour size and lymph vascular invasion, demonstrates the independent value of FOXA1 expression as a predictor of patient outcome in ERα-negative tumours. FOXA1 negativity is strongly related to breast cancer recurrence, this association being very close to statistical significance (FOXA1-negative vs. FOXA1-positive: hazard ratio = 7.02, 95% confidence interval = 0.92 to 53.37; *P *= 0.060). This analysis also confirmed that GATA-3 expression is not an important predictor of breast cancer recurrence in ERα-negative carcinomas (GATA-3-negative vs. GATA-3-positive patients: hazard ratio = 1.46, 95% confidence interval = 0.40 to 5.29; *P *= 0.559).

## Discussion

Several studies of global gene expression revealed high levels of FOXA1 often associated with the expression of ERα [6,35,36]. In addition, other gene whose expression has been highly correlated with ERα in breast cancer encodes the transcription factor GATA-3 [6,11,26,27]. Indeed, FOXA1, GATA-3 and ERα form a transcriptional circuit required for growth, differentiation and hormonal dependency of the lineage of mammary luminal cells [22,37]. Previous work using immunohistochemistry has shown that the expression of FOXA1 [10,31,33] and of GATA-3 [9,34,37] is in close association with ERα expression in breast cancer, highlighting their prognostic and predictive value in this malignancy. In fact, since these three proteins are components of a transcriptional network that dictates the phenotype of hormonal-dependent breast cancer [37], the study of their expression would improve our understanding of the ERα, FOXA1 and GATA-3 relationship in breast cancer patients.

In the present study the staining pattern of FOXA1 and GATA-3 in normal breast tissue is strikingly similar to that of ERα, which suggests the same cellular co-localization of these three cross-functional proteins. In the studied series, the expression of FOXA1 was inversely associated with clinicopathological features – namely, with tumour size, tumour grade, Nottingham Prognostic Index, lymph vascular invasion, lymph node stage and HER-2 overexpression – while its expression was directly associated with ERα, PR and the luminal A subtype. Thorat and colleagues, in a recent published study of 139 cases, did not demonstrate a significant association with tumour size, lymph node status or HER-2 [31]. Moreover, these authors also found an inverse association between FOXA1 and basal-like phenotype markers (namely, CK5 and CK14). Importantly, in our study we reinforced this inverse association between FOXA1 expression and the expression of P-cadherin or vimentin.

The requirement of this forkhead for optimal expression of at least 50% of ERα-regulated genes and oestrogen-induced proliferation was recently described [13], and our and other results may just represent the strong regulatory interdependency between ERα and FOXA1. Since ERα is one of the central genes for the regulation of growth/proliferation of mammary epithelia, and for the hormone-responsive phenotype of breast tumours [9], FOXA1 appears an important biological-regulatory factor with prognostic consequences in this setting. In fact, in the present study, FOXA1 expression was shown to be an important predictor of disease-free survival, in addition to the robust association with clinicopathological features. Interestingly, univariate analysis showed that the evaluation of FOXA1 expression has an important value in the assessment of the prognostic risk for breast cancer patient recurrence, with a magnitude of association similar to the observed for the classical prognostic factors, such as tumour size and lymph node stage, tumour grade, and ER and HER-2 expression. This finding is in line with previously published works, where both Badve and colleagues and Habashy and colleagues also demonstrated that FOXA1 expression is able to significantly predict a better survival for breast cancer patients [10,33], although the multivariate analysis showed that it is not an independent prognostic marker, exactly as shown for ER. These studies still suggest that ERα/FOXA1-expressing cells, after acquiring tumorigenicity, may promote selective clonal expansion, resulting in a specific subtype of breast cancer – the luminal subtype A. Thorat and colleagues also suggested that FOXA1 immunohistochemistry may be used as a marker for tumours pertaining to luminal subtype A breast cancer, which has an exceptionally good prognosis [31].

In contrast to FOXA1, GATA-3 failed the association with most of the clinicopathological features – the exception being an inverse association with HER-2 expression and tumour histological grade, although it was also directly associated with ERα and PR expression, as well as with tumours from the luminal A subtype. These results are partially in line with previous work from Mehra and colleagues, which found that low levels of GATA-3 expression were associated with higher tumour histological grade, positive lymph nodes, larger tumour size, negative ER expression and HER-2 overexpression [24]. In the present study we could not find an association between GATA-3 expression and lymph node status, in agreement with a recent cohort study from Voduc and colleagues comprising more than 3,000 invasive breast cancers [34]. Regarding the association between GATA-3 and ERα, 66% of the cases co-expressed these markers – which is a larger percentage than those previously described by Mehra and colleagues (46%) and by Voduc and colleagues (39%) [24,34].

Through the analysis of Kaplan–Meier survival curves it was not possible to demonstrate a significant association between GATA-3 expression and disease-free survival in this breast cancer series, which is in accordance with data from the large cohort study of Voduc and colleagues [34]. The univariate analysis confirmed this observation, although there is an association between the positivity for this marker and the better outcome for breast cancer patients.

Interestingly, the strength of the inverse association that was observed between GATA-3 and basal-like markers – namely, CK5, CK14, vimentin, EGFR and P-cadherin – suggests that GATA-3 can be important for the differentiation state of the malignant cells, where its presence, together with other differentiation involved partners, may drive the luminal profile of a malignant cell population within the tumour. Actually, this growth and differentiation role for GATA-3 in normal mammary epithelial cells has been already widely described [9,22,34,38]. Moreover, GATA-3-induced genes were found in the luminal cluster of gene expression studies, highlighting its putative ability to maintain a luminal differentiated phenotype [34].

In the past, several studies have shown that FOXA1 expression and GATA-3 expression are among the best predictors of ERα-positive status [9-12,26,31,33,34]. Additionally, some reports have proven that FOXA1 expression is able to significantly differentiate patients with a better survival within the luminal A subgroup, or even within the ERα-positive cohort (including luminal A and luminal B subtypes) [10,31]. These authors claim that FOXA1 can serve as a clinical marker for the luminal A subtype, and that its prognostic ability in these low-risk breast cancers may prove to be useful in clinical treatment decisions. In contrast, Habashy and colleagues did not find any clinical relevance in the immunohistochemical assessment of FOXA1 in breast cancer routine practice [33], since it was not able to stratify ER-positive (luminal-like) tumours into clinically significant subgroups.

Although never assessed, the difference between these studies can be possibly due to the endocrine and chemotherapy administered to the different series of patients, which can block the ERα-associated pathways and confound interpretation of the results. Moreover, since ERα, FOXA1 and GATA-3 show an intrinsic high correlation between themselves, the prognostic and predictive values of these markers may simply reflect this high expression association and the described biological interactions.

In order to study whether there was a prognostic value for the expression of these two transcription factors in the absence of ERα expression, we therefore decided to perform an exploratory subgroup analysis in a cohort of ERα-negative patients. The aim was to test, for the first time, the possible utility of FOXA1 and/or GATA-3 as classifiers for breast cancer recurrence in this high-risk subset of patients, revealing a stratification of ER-negative tumours with different biological behaviours. Interestingly, only FOXA1-positive expression showed a clear protective effect for breast cancer relapse in this cohort of patients with poor prognosis. Patients with loss of FOXA1 tumour expression showed an increased risk for breast cancer recurrence compared with the patients that were positive for this marker. The relative risk estimate was higher than that calculated for HER-2 positivity, which is a well-known prognostic factor in hormone-independent breast carcinomas. Moreover, the multivariate analysis, including the tumour size and lymph node status, demonstrated the independent value of FOXA1 as a predictor of patient outcome in ERα-negative tumours.

In conclusion, our results confirmed the strong association between ERα and FOXA1 in breast cancer and confirmed the role of FOXA1 as a significant breast cancer predictor of good outcome in univariate analysis, directly associated with luminal A and inversely associated with basal-like subtype of breast cancer. GATA-3 was neither a predictor for breast cancer disease-free survival nor a prognostic marker, but was shown to be an important and robust luminal differentiation marker, even stronger than FOXA1. Based on these findings, the expression assessment of FOXA1 and GATA-3 in breast cancer patients can provide important clinical information – not only regarding the favourable prognostic nature and tumour behaviour, but the expression can also constitute an important tool to define and assess the luminal A subtype in breast cancer. We demonstrated that FOXA1 expression also has an important role as breast cancer predictor of good outcome in ER-negative breast carcinomas.

Based on our results, we can consider that the expression of FOXA1, as an ER-associated gene, may be important to the hormone-responsive phenotype of breast cancer, regardless of the tumour ER status. The absence of FOXA1 in luminal/ER-positive breast cancer patients may contribute to identify the 30% of ER-positive tumours that are not hormone responsive. Additionally, because of the known cross-talk and functional network between FOXA1 and the regulation of ERα and its downstream targets, the expression of FOXA1 in ER-negative breast cancer patients may represent the existence of an alternative oestradiol-independent response pathway, which may allow the 5 to 15% of ER-negative tumours to become responsive to endocrine-driven therapies. The clinical implication of these findings requires a larger prospective cohort, especially to evaluate the value of FOXA1 in the therapeutic response setting. Nevertheless, the current study already represents an important step forward in the overview the ER-negative type of tumours, with putative future benefit for staging and treatment of these patients.

## Conclusions

Current challenges in oncology include prediction of tumour behaviour and selection of effective therapy for individual treatment based on molecular targets. In breast cancer, ERα expression alone has been used to guide systemic therapy and to estimate patient prognosis. Not all ER-positive carcinomas, however, show comparable prognosis or react similarly to anti-hormonal therapy, and some ER-negative tumours curiously respond to therapy. This clinical evidence demonstrates that breast carcinomas are extremely heterogeneous, emphasizing the need for improving the molecular classification within tumours to better predict their clinical behaviour and the patient's response to current therapies.

The identification of transcription factors that control the ERα pathway provide an opportunity to identify specific subsets of patients that will have a good prognosis, as well as who will benefit from endocrine treatment. In the present work, we studied FOXA1 and GATA-3 expression in order to evaluate whether the proteins would predict the recurrence behaviour of breast cancer patients. We verified that patients harbouring FOXA1-positive and ER-negative tumours show a better disease-free survival, demonstrating the clinical importance of these two biomarkers in breast cancer molecular classification and prognosis. The analyses showed that FOXA1 and ERα should be used together in order to subclassify breast carcinomas and to predict the outcome of breast cancer patients.

## Abbreviations

CK: cytokeratin; EGFR: epidermal growth factor receptor; ER: oestrogen receptor; FOXA-1: forkhead box A1; GATA-3: GATA binding protein 3; HER-2: human epidermal growth factor receptor 2; PBS: phosphate-buffered saline; P-cadherin: placental cadherin; PR: progesterone receptor.

## Competing interests

The authors declare that they have no competing interests.

## Authors' contributions

AA, BS, DV and NLo carried out all the immunoassays and general laboratory work. FM, VC and FS were the pathologists who revised and classified all cases. NLu, JB and SC performed the statistical analysis. AA and JP contributed to the conception and design, analysis and interpretation of data. AA, JP, FM, EWL and FS were involved in drafting the manuscript and revising it critically for important intellectual content. All authors read and approved the final manuscript.
